# Effects of Diet Xylooligosaccharide Supplementation on Growth Performance, Carcass Characteristics, and Meat Quality of Hu Lambs

**DOI:** 10.3390/foods14040656

**Published:** 2025-02-15

**Authors:** Jiaxin Yang, Wanhang Jia, Binglei Zhang, Saiyi Sun, Xueru Dou, Qiujue Wu, Yuqin Wang, Yuanxiao Li, Wenfeng Ma, Guoyan Ren, Xiaoyin Zhang, Yang Wang

**Affiliations:** 1 College of Animal Science and Technology, Henan University of Science and Technology, Luoyang 471023, China; yang18317549549@163.com (J.Y.); jwh980323@163.com (W.J.); z18790606037@163.com (B.Z.); sunsaiyi2023@163.com (S.S.); 13343795208@163.com (X.D.); wuqiujue@163.com (Q.W.); lyx8023@yeah.net (Y.L.); a113boy@163.com (W.M.); zhangxiaoyin0426@163.com (X.Z.); wangyocean@163.com (Y.W.); 2 College of Food & Bioengineering, Henan University of Science and Technology, Luoyang 471023, China; renguoyan@163.com

**Keywords:** xylooligosaccharides, growth performance, carcass characteristics, meat quality, correlation analysis, Hu lambs

## Abstract

In this study, we examined the effect of xylooligosaccharide (XOS) supplementation on the growth performance, carcass characteristics, and meat quality of Hu lambs. In total, 60 Hu lambs (two months old and weighing 17.32 ± 0.81 kg) were randomly assigned to four treatment groups, each with three replicates and five lambs per replicate. The lambs were fed basal diets supplemented with 0, 1.5, 3, or 4.5 g/kg XOSs in a basal diet for 60 days, with the groups designated XOS0%, XOS1.5%, XOS3%, and XOS4.5%, respectively. The results revealed, compared to theXOS0% group, the XOS3% group presented a lower F:G during 31 to 45 d (*p* = 0.06). By the 60th day, the body length indices of groups XOS3% and XOS4.5% increased compared to the XOS0% group, with a significant increase observed in group XOS4.5% (*p* < 0.05). Additionally, the GR values of the XOS1.5%, XOS3%, and XOS4.5% groups increased significantly, and the rumen fluid pH values of the XOS3% and XOS4.5% groups increased significantly (*p* < 0.01). The crude fat content in the XOS1.5% and XOS4.5% groups were significantly lower (*p* < 0.05). The hardness, adhesiveness, elasticity, cohesiveness, and chewiness of the mutton in the XOS1.5%, XOS3%, and XOS4.5% groups were increased, although the differences were not statistically significant (*p* > 0.05). Correlation analysis indicates that there is a significant correlation between growth performance, carcass traits, and meat quality (*p* < 0.05). The factors influencing meat quality originate from the growth period and the slaughtering phase, which can be attributed to the effects of xylooligosaccharides. In conclusion, XOS had positive effects on the growth performance, carcass characteristics, and meat quality of Hu lambs. The comprehensive effect of group XOS3% was best. Considering the production cost, the 3 g/kg XOSs is identified as the optimal supplementation level for sheep.

## 1. Introduction

Chinese people have been consuming mutton since ancient times. With economic development and improvements in living standards, the demand for better mutton quality among consumers has increased [1]. In the past, antibiotics have been commonly employed for the treatment of animals and to enhance their growth. However, the excessive use of antibiotics has led to a plethora of safety concerns, including increased antibiotic resistance, environmental contamination, and drug residues, all of which pose potential risks to animal health, human safety, and human health [2,3]. With the establishment of national policies to reduce and prohibit antibiotic usage, the conflict between sheep health and food safety issues for humans is increasing, and scientists are searching for alternative antimicrobial products to ensure animal health and guarantee a high-quality and safe meat product [4,5].

In recent years, various antibiotic alternatives have been developed domestically, such as acidifiers, functional oligosaccharides, enzyme preparations, trace element additives, compound enzyme preparations, traditional Chinese medicine extracts, and intestinal probiotics [6,7,8]. These alternatives can exert effects similar to those of antibiotics but also show varying levels of resistance. Functional oligosaccharides have good resistance substitution effects [9,10,11]. They can not only promote the proliferation of beneficial bacteria in the intestine but also inhibit the adhesion of pathogens in the intestine [12,13]. They can also enhance immunity and antioxidant capacity, thus promoting the growth and development of animals [14,15]. Most importantly, they are non-toxic, free of side effects, and do not leave harmful residues in animals, further ensuring food safety [16].

Xylooligosaccharides (XOSs) are among the most popular types of functional oligosaccharides and also a prebiotic with high nutritional levels and health benefits [17]. Compared to other oligosaccharides, they have several key advantages, such having as a small amount of additives, being not easily decomposed by animal digestive enzymes, and highly selectively promoting the proliferation of beneficial bacteria such as *Bifidobacterium* [18,19,20]. XOSs are essential alternatives to antibiotics in the large-scale breeding of animals, with growth performance, carcass traits, and meat quality being closely related to the effects of XOS. Multiple studies have demonstrated that XOS plays an active role in improving the antioxidant capacity, immune function, and intestinal tissue morphology of broilers. The incorporation of XOS into feed has been shown to enhance feeding behavior, growth performance, and meat quality in broilers [21,22]. Wang et al. [23] observed that broilers receiving a diet supplemented with 100 mg/kg XOS and 600 mg/kg IAPS exhibited increased average daily gain and improved feed efficiency, along with potential benefits to intestinal morphology and barrier function. Rao et al. [21] demonstrated that supplementing the basal diet with 150 and 450 mg/kg XOSs significantly boosted average daily gain, reduced feed conversion ratio and abdominal fat percentage, and elevated serum IgA and IgG levels, particularly in the 300 mg/kg XOS group. This supplementation also increased the relative abundance of short-chain fatty acid-producing bacterial genera. Overall, XOS addition to broiler diets enhances growth performance, promotes gut health by strengthening gut barrier function and modulating cecal microbiota diversity, and positively impacts immune function. Li et al. confirmed that XOS can enhance feeding behavior and improve growth performance in broiler chickens during the grower stage by increasing average daily gain. Moreover, XOS supplementation improves post-slaughter muscle pH (of fresh meat) and reduces cooking loss, with the most pronounced effects observed at a dosage of 300 mg/kg. [24]. Additionally, one study by Pang et al. [25] showed that XOS can improve growth performance in weaning piglets by enhancing antioxidant capacity, immune function, and beneficial bacterial counts, with an optimal supplementation level of 1.5%. Abasubong et al. [26] demonstrated that XOS can improve lipid metabolism in carp by modulating plasma lipid levels and gene expression related to lipid metabolism. This includes upregulating lipoprotein lipase and downregulating carnitine palmitoyl transferase I, peroxisome proliferator-activated receptor α, acyl-CoA oxidase, and CD36. It is recommended to add 10–20 g/kg XOS to the feed. Previous studies have primarily focused on piglets, broiler chickens, and aquatic animals, while the mechanism of action of XOS on sheep remains unclear.

Growth performance, carcass traits, and meat quality serve as key metrics for evaluating the efficiency and quality of food animal production [27,28]. Growth performance includes metrics like average daily gain (ADG), feed conversion ratio (FCR), and body measurement index, which directly indicate an animal’s growth rate, feed efficiency, and physical development. Carcass traits pertain to the quality of the carcass and the distribution of meat and fat, which are essential criteria for assessing an animal’s meat value and determining its commercial worth and market competitiveness. Meat quality comprises attributes like color, tenderness, pH, texture, and nutritional content, which are directly linked to meat product sales and consumer preferences. Thorough research on these indicators is essential for evaluating animal health and welfare and optimizing breeding and management practices, thereby enhancing the quality and market competitiveness of food animal products and fostering the sustainable development of the livestock sector.

To clarify how xylooligosaccharides (XOSs) work in sheep, we selected XOS levels of 0, 1.5, 3, and 4.5 g/kg based on the findings from these previous studies and the nutritional requirements of Hu lambs. This study will investigate the effects of varying concentrations of XOS on the growth performance, carcass characteristics, and meat quality of Hu lambs, while determining the optimal level of XOS supplementation in sheep production. Additionally, potential correlations among various production performances will be analyzed to explore effective approaches for promoting the growth and development of sheep, providing a theoretical foundation for subsequent research.

## 2. Materials and Methods

### 2.1. Animal Model and Management

All animal experiments were conducted from July to September 2023 at the breeding sheep farm of Henan Kunyuan Agriculture and Animal Husbandry Technology Co., Ltd. in Ruzhou City, Henan Province, China. The experiments were approved by the Institutional Animal Care and Use Committee of Henan University of Science and Technology (HAUSTEAW-2021-C00227).

The animals were housed individually in enclosures with slatted floors for waste removal. During the transition period of the experiment, the animals were dewormed using ivermectin and vaccinated against peste des petits ruminants, sheeppox, and triple-four prevention diseases. The animals were fed twice daily at 8:00 a.m. and 5:00 p.m. using a Total Mixed Ration (TMR). The remaining feed was weighed the following morning each day, before feeding the animals. The daily feed allocation was adjusted based on actual consumption to ensure optimum feeding and free access to clean water. During the transition period, all experimental animals were fed a basal diet. At the beginning of the experiment, the animals were fed according to their designated group assignments. The experiment lasted 60 days, split into a 15-day preliminary period and a 45-day formal experimental period.

A single-factor experimental design was adopted, and the experiment was conducted using 60 male Hu lambs (about two months old and weighing about 17.32 ± 0.81 kg for fattening). The animals were randomly divided into four groups, with three replicates per group, each consisting of five sheep. The XOS0% group was fed a basal diet, whereas the XOS1.5%, XOS3%, and XOS4.5% groups were supplemented with 1.5, 3, and 4.5 g/kg XOS, respectively, in their basal diets. The basal diet was formulated according to the nutritional requirements specified in “Nutrient Requirements of Meat Sheep” (NY/T 816—2021); the composition and nutritional levels are listed in Table 1. XOS was purchased from Henan Kornbo Agricultural Technology Co., Ltd. (Zhengzhou, China). The content was 20%, and the remaining components were cellulose, hemicellulose, and lignin.

### 2.2. Growth Performance

On the mornings of day 1 and day 60 of the experiment, the test animals were weighed after they fasted 12 h overnight to determine the initial and final body weights, respectively. Throughout the trial, the Hu lambs were fasted and weighed every 15 days to calculate the average daily weight gain (ADG) for the periods 1–15, 16–30, 31–45, and 46–60 days, as well as for 1–60 days (the whole period) [29]. The daily feed intake and the amount of leftover feed were recorded every day to calculate the average daily feed intake (ADFI). The feed to gain ratio (F:G) was calculated based on the ADG and ADFI. Body measurements were also conducted while weighing to calculate the body index [30]. The formulae used to calculate the body indices are as follows:

Body length index (%) = 100 × body length/body height;

Body trunk index (%) = 100 × chest circumference/body length;

Chest circumference index (%) = 100 × chest circumference/body height;

Metacarpal circumference index (%) = 100 × metacarpal circumference/body height.

### 2.3. Carcass Characteristics

On the 60th day of the experiment, three sheep were randomly selected from each group for slaughter, excluding extreme outliers, totaling 12 sheep with an average body weight of 26.64 ± 1.63 kg. Before slaughter, the lambs were subjected to a 12 h fast, abstaining from both food and water. The lambs were processed to assess various slaughter-related parameters, which included the following measurements: pre-slaughter weight, net meat weight, bone weight, and carcass weight (defined as the weight of the entire body, including kidneys and kidney fat, after removal of the skin, head, internal organs, and limbs below the knee joint of the forelimb and toe joint of the hindlimb, measured after a 30 min rest period). Additionally, the slaughter rate (the ratio of carcass weight to the live weight of the animal after a 12 h fast), meat yield, meat-to-bone ratio, tail fat, rumen pH, longissimus dorsi muscle area, and GR value (measured as the tissue thickness at the GR site, located between the 12th and 13th ribs and 11 cm from the midline of the dorsal spine, serving as an indicator of carcass fat content) were determined.

### 2.4. Meat Quality Analysis

Specimens of the longissimus dorsi muscle were gathered from each sheep, and these meat specimens were stored in a refrigerator at 4 °C for a period of 72 h to evaluate their physicochemical attributes and palatability. The physicochemical attributes encompassed moisture level, lipid content, pH value, cooking loss, shear force, and the hue of the mutton, while the palatability aspects covered textural profiles.

The moisture content was determined according to GB 5009.3 using an LBAO-150H ventilated oven (Stu Kai Instrument Equipment Co., Ltd., Seki City, Japan). The fat content was measured according to GB 5009.6 using a fat analyzer (Hannon SOX406, Jinan, China). The muscle pH was assessed based on NY/T 2793-2015 using a pH meter (Testo 205). The water-holding capacity was evaluated according to NY/T 2793–2015 with an HWS-24 electric heating constant-temperature water bath (Shanghai—Heng Science Instrument Co., Ltd., Shanghai, China). The shear force was determined by placing about 50 g of the meat sample in a cooking bag, removing the air from the bag to ensure that the meat surface was in close contact with the bag, sealing the bag, and heating it in a 71 °C constant-temperature water bath for 30 min. After the sample was cooled to room temperature and stored overnight in a refrigerator at 4 °C, the meat was cut into 1.0 cm × 1.0 cm × 1.5 cm pieces perpendicular to the muscle fibers and examined using a tenderness meter (C-LM3B). The color stability of the mutton was measured using a color difference meter (CM-600D), with four random points selected on the surface of each sample, and the average value of the four measurements was calculated [31].

Textural characteristics were assessed by taking about 50 g of the meat sample following the same procedure as for shear force determination and then cutting the cooled meat into 2.0 cm × 2.0 cm × 1.5 cm pieces along the muscle fibers. These samples were then analyzed using a texture analyzer (TA-XT plus) [32].

### 2.5. Statistical Analysis

All experimental data were subjected to analysis using SPSS 26.0 software (SPSS Inc., Chicago, IL, USA). Group differences were assessed via one-way ANOVA, succeeded by Duncan’s multiple range test. Results were presented as the mean ± standard error of the mean (SEM). Statistical significance for differences was set at *p* < 0.05, while 0.05 < *p* < 0.10 denoted a trend. Spearman’s rank correlation coefficients were employed to evaluate the relationships between parameters.

## 3. Results

### 3.1. Growth Performance

The growth performance results are presented in Table 2. XOS supplementation did not significantly influence body weight, ADG, ADFI, or the F:G in Hu lambs. However, a decrease in F:G was observed from days 31–45 with increasing XOS, with the XOS3% group exhibiting the lowest F:G (*p* = 0.06). From days 16 to 30, the control group presented a greater ADG than the other three experimental groups. However, from days 31 to 60, the ADG of the three experimental groups surpassed that of the control group, with an increase in the later phase. Over the entire experimental period, the ADG of the four groups showed no significant differences (*p* > 0.05). Additionally, no significant differences were found in the ADFI among the four experimental groups across all stages (*p* > 0.05).

### 3.2. Body Measurement Index

As shown in Table 3, on the first day of the experiment, the chest circumference indices of the XOS1.5% and XOS3% groups were significantly lower than that of the control group (*p* < 0.05). The body length index was also lower than that of the control group, but the difference was not significant (*p* = 0.084). By the 60th day, the body length indices of the XOS3% and XOS4.5% groups were greater than those of the control group, with a significant increase recorded in the XOS4.5% group (*p* < 0.05). Compared to that in the control group, the body trunk index was lower in the three experimental groups (*p* = 0.066); the chest circumference indices of the XOS1.5% and XOS3% groups were lower, whereas that of the XOS4.5% group was higher (*p* = 0.085).

### 3.3. Carcass Characteristics

As shown in Table 4, the GR values of Groups XOS1.5%, XOS3%, and XOS4.5% were significantly greater than those of the XOS0% group (*p* < 0.01), but the GR values of Group XOS3% were lower than those of Groups XOS1.5% and XOS4.5%. Additionally, the pH of the rumen fluid of the XOS3% and XOS4.5% groups was significantly greater than that of the XOS0% group and XOS1.5% group (*p* < 0.01). The tail fat weight of the XOS1.5% and XOS3% groups was lower, whereas that of the XOS4.5% group was higher, compared to that of the XOS0% group (*p* = 0.094).

### 3.4. Mutton Quality Characteristics

As shown in Table 5, the fat content in the muscle of the XOS1.5%, XOS3%, and XOS4.5% groups was lower than that in the XOS0% group, with the XOS1.5% and XOS4.5% groups showing a significant decrease (*p* < 0.05). Additionally, compared to the muscle pH in the XOS0% group, the pH of the XOS1.5% and XOS4.5% groups was lower, whereas that of the XOS3% group was higher (*p* = 0.093). No significant differences were recorded for the other indices (*p* > 0.05). However, the XOS4.5% group had the highest muscle moisture content and shear force and the lowest fat content, muscle pH, and cooking loss. The XOS3% group had the highest muscle pH and yellowness (b*) and the lowest redness (a*) and crude protein content.

### 3.5. Textural Properties

As indicated in Table 6, the XOS1.5%, XOS3%, and XOS4.5% groups presented greater hardness, adhesiveness, springiness, gumminess, and chewiness than the control group (*p* > 0.05), whereas the cohesiveness and resilience were lower (*p* > 0.05). Specifically, the XOS4.5% group presented the highest values for hardness, adhesiveness, and gumminess, the XOS3% group presented the lowest cohesiveness, and the XOS1.5% group presented the highest springiness and chewiness. However, there were no significant differences (*p* > 0.05).

### 3.6. Correlation Analysis

**Figure 1:**
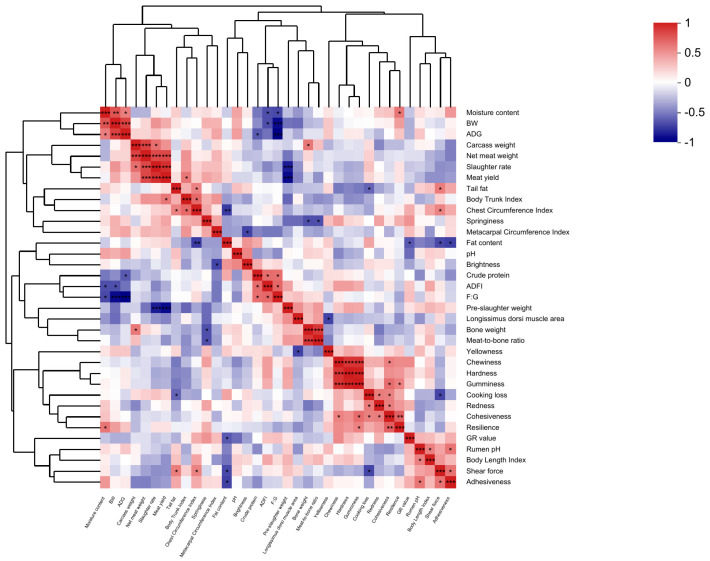
Correlation analysis of various production performances in Hu lambs. * *p* < 0.05: Significant correlation; ** *p* < 0.01: Highly significant correlation; *** *p* < 0.001: Very highly significant correlation.

A correlation coefficient matrix heat map provides a visual representation of the linear associations among different production traits. As illustrated in Figure 1, the ADG of Hu lambs exhibited a significant positive correlation with moisture content and BW (*p* < 0.05), while a significant negative correlation was observed with F:G (*p* < 0.05). The F:G ratio demonstrated a significant positive correlation with crude protein content and ADFI (*p* < 0.05) and a significant negative correlation with moisture content, BW, and ADG (*p* < 0.05). The slaughter rate showed a significant positive correlation with carcass weight, net meat weight, and meat yield (*p* < 0.05), while being significantly negatively correlated with preslaughter weight (*p* < 0.05). Meat yield was significantly positively correlated with net meat weight, slaughter rate, and body trunk index (*p* < 0.05) and significantly negatively correlated with preslaughter weight (*p* < 0.05). Tail fat was positively associated with the chest circumference index and shear force (*p* < 0.05) and negatively associated with cooking loss (*p* < 0.05). The area of the longissimus dorsi muscle was significantly negatively correlated with yellowness (*p* < 0.05), while bone weight was significantly positively associated with carcass weight and the meat-to-bone ratio (*p* < 0.05) and significantly negatively correlated with springiness (*p* < 0.05). Rumen pH displayed a significant positive correlation with body length index and adhesiveness (*p* < 0.05). The chest circumference index was positively associated with meat yield, tail fat, and shear force (*p* < 0.05) and negatively associated with fat content (*p* < 0.05). Springiness was significantly negatively correlated with bone weight and the meat-to-bone ratio (*p* < 0.05). Fat content exhibited a significant negative correlation with the GR value, shear force, and adhesiveness (*p* < 0.05). Crude protein was positively associated with ADFI and F:G (*p* < 0.05), while being negatively correlated with ADG (*p* < 0.05). Cooking loss showed a significant positive correlation with redness and cohesiveness (*p* < 0.05) and a significant negative correlation with tail fat and shear force (*p* < 0.05). Redness was positively associated with cooking loss and cohesiveness (*p* < 0.05). Shear force was significantly positively correlated with tail fat, chest circumference index, and adhesiveness (*p* < 0.05) and negatively correlated with fat content and cooking loss (*p* < 0.05). Chewiness was significantly positively correlated with hardness, gumminess, and cohesiveness (*p* < 0.05). Gumminess was positively associated with chewiness, hardness, cohesiveness, and resilience (*p* < 0.05). Cohesiveness showed a positive correlation with chewiness, hardness, gumminess, cooking loss, redness, and resilience (*p* < 0.05). Resilience was positively associated with moisture content, gumminess, and cohesiveness (*p* < 0.05). Adhesiveness was significantly positively correlated with rumen pH and shear force (*p* < 0.05) while being negatively correlated with fat content (*p* < 0.05).

## 4. Discussion

The ADG, ADFI, and F:G ratio can reflect the growth rate, feed intake, and feed utilization rate of animals, respectively. When the F:G ratio is lower, the amount of feed required per unit weight gain is smaller, indicating higher feeding efficiency. Body measurement indicators can be used to represent the size of various parts of the body surface of livestock, and body measurement index can be used to represent the degree of relative development of the body [33]. Several studies have shown that XOS can modulate the gut microbiota upon ingestion, enhancing the presence of probiotics and diminishing pathogenic bacteria. This may promote muscle growth in animals, as intestinal health directly influences nutrient absorption. Li et al. [24] showed that administering XOS or FLA to chickens significantly improved the ADG. Moreover, administering XOS to chickens, in addition to a wheat-rye-based diet, significantly improved the FCR. The same results have been reported in pigs. There are few studies on the effects of XOS in ruminants. Dong et al. found that dietary supplementation with XOS and EXE significantly improved milk production performance, nutrient digestibility, and energy utilization efficiency in lactating Jersey cows, while reducing enteric CH4 emissions. Their study indicates that XOS and EXE can serve as effective mitigation strategies to enhance production performance and reduce environmental impact. Further research is needed to validate the long-term effects and mechanisms of action in dairy cows. This study can provide a reference for the application of XOS in ruminant nutrition by investigating its effects on sheep. This study showed that different levels of XOS did not significantly affect the ADG, ADFI, FCR, or most body measurement indices of Hu lambs at all stages. However, in the experiment, compared to those of the XOS0% group, the body length indices of the XOS3% and XOS4.5% groups tended to increase, and those of the XOS4.5% group increased significantly (*p* < 0.05). Additionally, the ADG of the XOS3% group increased to a greater extent throughout the entire period, and the F:G ratio showed a decreasing trend throughout the entire period. This finding is generally consistent with previous research outcomes. However, it is noteworthy that the rumen is a unique and powerful digestive organ in ruminants, capable of digesting coarse feed that is difficult for monogastric animals to break down, and producing volatile fatty acids (VFAs), which provide 60% to 70% of the energy for ruminants [34]. The nutritional effects of XOS may be related to the structure of the rumen microbiota. In this experiment, as the level of XOS increased, the pH of the rumen fluid showed an increasing trend, indicating that XOS could improve the productive performance of ruminants by altering rumen fermentation, affecting the production of VFAs, stimulating the growth of rumen microorganisms, and increasing the rate of fiber degradation.

Carcass characteristics are important indicators of meat quality in livestock and poultry [27]. In this study, they directly reflected whether the addition of XOS affected the meat production of Hu lambs. The results showed that different levels of XOS can increase the GR value and pH of the rumen fluid. The GR value reflects the amount of body fat, and a higher GR value indicates a greater fat content in the carcass of sheep [35]. Li et al. [36] found that the increase of GR value will increase the fat content, especially the intramuscular fat (IMF), which usually improves the tenderness and juiciness of meat and helps to improve the water retention ability and taste of meat. At the same time, it can also promote the synthesis of flavor precursors, so as to improve the overall flavor of meat. The normal pH of the rumen is 6.2–6.8 [37], and the pH of all four treatment groups in this study was below this range. However, as the level of XOS increased, the pH of the rumen fluid increased, with the XOS3% and XOS4.5% groups showing a significant increase. This occurred probably because during the fattening period, male lambs require a lot of energy, and their diet contains a greater proportion of grain feed. Grain feed is rich in monosaccharides, starch, and other carbohydrates, which can be easily fermented in the rumen and can quickly produce volatile fatty acids (VFAs). When animals consume excessive amounts of grain feed, many VFAs are produced in the rumen [38]. Failure to remove these VFAs can decrease the pH of the rumen to about six.

Meat color, pH, and cooking loss are the main factors that determine the physicochemical properties of meat, with meat color being the most intuitive indicator of meat quality. The L*, a*, and b* values are indicators for evaluating meat color: the smaller the L* and b* values, and the larger the a* value, the better the meat color [39]. The muscle pH is an important indicator for measuring meat quality; the muscle pH value affects water loss and meat color. The higher the muscle pH value, the better the water-holding capacity, the softer the muscle, and the better the freshness and meat color [40,41]. Shear force is an indicator for measuring muscle tenderness, the lower the shear force, the more tender the meat [42]. Protein and fat contents are important factors affecting meat quality and are closely monitored indicators in studies on meat quality. The nutritional value of meat is related to its protein content. The differences in meat flavor mainly originate from the decomposition and oxidation of fats [43]. Li et al. [44] reported XOS was able to reduce cooking losses in broilers while increasing the pH of pectoral and thigh muscles. Muscle pH has an important impact on water retention, and a higher pH may help reduce cooking losses. In this study, no significant differences were recorded in moisture content, cooking loss, shear force, L* value, a* value, b* value, muscle pH, or crude protein content among the four experimental groups. However, compared to the XOS0% group, the XOS4.5% group presented the highest moisture content and the lowest degree of cooking loss. The L* values in groups XOS1.5% and XOS3% were lower, and the b* values in groups XOS3% and XOS4.5% were lower. The XOS3% group had the highest muscle pH, indicating that the addition of XOS to the diet improved meat color to some extent. The crude fat content in the XOS4.5% group was significantly lower than that in the control group, probably because XOS promoted the proliferation and metabolism of lactic acid bacteria and Bifidobacterium, and the active metabolic products improved the antioxidant capacity and activated lipase in the body, thereby inhibiting oxidation of the body and promoting fat breakdown.

The tenderness of the meat is related to textural parameters such as hardness, chewiness, and springiness [45,46]. Hardness is associated with the muscle fiber structure and protein content of meat products; tougher meat may indicate a denser muscle fiber structure or higher protein content [47]. Adhesiveness, springiness, cohesiveness, and chewiness are associated with the palatability of meat products; higher elasticity and appropriate levels of adhesiveness, cohesiveness, and chewiness imply a better mouthfeel [48,49]. Zhang et al. [50] demonstrated that xylooligosaccharides have a significant inhibitory effect on the textural properties (elasticity and chewiness) of shrimp, and in addition, they can maintain a higher content of myofibrillar protein and Ca^2+^-ATPase activity. Wu et al. [51] discovered that xylooligosaccharides at an appropriate level (1.0 mg/kg) have positive effects on fish growth, feed efficiency, intestinal health, muscle texture (with the highest hardness, gumminess, and chewiness), and intestinal microbiota, but excessive supplementation may lead to negative effects. These effects may be achieved by regulating intestinal health, muscle protein stability, and the composition of the intestinal microbiota. This study showed that XOS did not significantly affect textural properties, but compared to those of the XOS0% group, hardness, adhesiveness, springiness, gumminess, and chewiness tended to increase in all experimental groups, whereas cohesiveness and resilience tended to decrease. By comprehensively analyzing seven indicators (hardness, adhesiveness, springiness, gumminess, cohesiveness, chewiness, and resilience), we concluded that adding XOS to the diet can improve some textural characteristics to a certain extent and positively affect the quality of mutton.

Determining the relationships between various production indicators in Hu lambs via correlation analysis may contribute to the development of breeding and production activities. Shen et al. [52] observed when the red and intramuscular fat content of the lamb increases, the shear force of the meat decreases. Xu et al. [53] found that muscle quality can be improved by reshaping the gut microbiota and altering SCFA levels. Schumacher et al. [54] reported that IMF (intramuscular fat) content is positively correlated with the juiciness, wetness, and tenderness of meat, and reasonable IMF content is helpful to improve the quality and flavor of meat. We found significant correlations between growth performance, carcass characteristics, and meat quality. Specifically, fat content was significantly negatively correlated with the GR value, shear force, and adhesiveness (*p* < 0.05), which may be attributed to the fact that a relatively high fat content can increase the WHC (Water Holding Capacity) of meat, thus reducing cooking loss and increasing tenderness [55]. Additionally, an increase in fat content may decrease the density of muscle fibers, leading to a decrease in shear force and a softer and smoother texture of the meat. The crude protein content was significantly positively correlated with the ADFI and F:G ratio (*p* < 0.05), indicating a close relationship between protein intake and the growth performance of the animals. In contrast, a significant negative correlation with ADG occurred, probably due to an increase in muscle synthesis and deposition of lower levels of fat during the rapid growth phase, resulting in alterations in meat quality. In practical production, the optimization of animal production performance and meat quality can be achieved early on by adjusting feed formulations and husbandry management practices, thereby increasing economic benefits and meeting consumer demands for high-quality meat products. By analyzing these correlations, we can better understand the factors influencing meat quality and implement corresponding measures to improve the sensory and nutritional characteristics of meat. In addition, this correlation may involve lipid metabolism, including effects on fat absorption and digestion, patterns of fat deposition, and the quality of fat and protein in muscle. This provides direction for further exploration of the underlying molecular mechanisms.

The results of this study indicate that XOS supplementation has the potential to improve the growth performance and meat quality of Hu lambs. Specifically, the XOS3% group (supplemented with 3 g/kg XOS) showed the highest average daily gain (ADG) and a lower feed-to-gain ratio (F:G), suggesting improved feed efficiency. These improvements can translate into economic benefits for livestock producers by reducing feed costs and increasing the market value of the meat. Additionally, the observed improvements in meat quality, such as increased tenderness and reduced cooking loss, can enhance consumer satisfaction and potentially command higher prices in the market. The optimal dosage of 3 g/kg XOS, as identified in this study, balances the cost of supplementation with the benefits gained, making it a practical and economically viable option for sheep production.

## 5. Conclusions

This study discovered that xylooligosaccharides (XOSs) have certain positive effects on the growth performance, carcass traits, and meat quality of Hu lambs. XOS can reduce feed-to-gain ratio (F:G) during a specific period (31 to 45 d), promote body length development, and also increase GR values and ameliorate textural properties in lambs. XOS3% and XOS4.5% exhibited better outcomes, and considering production costs, the XOS level corresponding to Group XOS3% was determined to be optimal, with 3 g per kilogram of XOS being the most suitable additive amount in sheep production. Furthermore, the correlation analysis of various production indicators indicated that XOS could influence the edible quality of meat by improving the physical development and feed conversion rate of Hu sheep, altering the physicochemical properties and basic nutritional components of muscle.

## Figures and Tables

**Table 1:** Composition and nutrient levels of the basal diets (air-dry basis).

Ingredients		Nutritional Components ^2^ (%)	
Corn	63.00	Dry matter	79.79
Soybean meal	16.00	Moisture	13.5
Wheat bran	8.00	Crude ash	4.51
Hsin cathepsin	3.00	Crude protein	8.83
Baking soda	1.50	Ether extract	2.69
MgO	0.80	NDF	16.39
NaCl	1.20	ADF	6.85
Plant oil	0.80	Crude fiber	1.86
Mold inhibitor	0.30	Starch	49.91
Amino Acid Modulator YSS-3	0.40	ADL	1.28
5% lamb robustness premix ^1^	5.00	Calcium	1.97
Total	100.00		

^1^ The premix contained: Fe—60 mg, Zn—50 mg, Cu—10 mg, I—0.6 mg, Mn—44 mg, Se—0.25 mg, Co—0.21 mg, VA 2800 IU, VE 100 IU. ^2^ Nutrient levels are measured using a near infrared analyzer (ZEISS, Germany).

**Table 2:** Effects of XOS on growth performance of Hu lambs.

Item	Feeding Stage	Group				SEM ^1^	*p*-Value
		XOS0%	XOS1.5%	XOS3%	XOS4.5%		
BW ^2^, kg	1 d	17.50	17.33	17.17	17.50	0.391	0.802
	15 d	19.42	19.28	19.03	19.10	0.591	0.911
	30 d	22.13	21.13	20.67	20.75	0.621	0.149
	45 d	23.80	23.07	23.62	23.68	0.948	0.869
	60 d	26.65	26.35	26.60	26.82	0.727	0.932
ADG ^3^, g	1 to 15 d	136.90	139.29	133.33	114.29	24.643	0.740
	16 to 30 d	194.05	132.14	116.67	117.86	33.619	0.146
	31 to 45 d	119.05	138.10	210.71	209.52	41.394	0.120
	46 to 60 d	203.57	234.52	213.10	223.81	50.233	0.932
	1 to 60 d	155.08	152.82	159.89	157.91	12.397	0.942
ADFI ^4^, kg	1 to 15 d	0.87	0.89	0.90	0.88	0.045	0.896
	16 to 30 d	1.86	1.89	1.87	1.86	0.040	0.892
	31 to 45 d	1.91	1.92	1.88	1.84	0.052	0.485
	46 to 60 d	1.76	1.69	1.71	1.73	0.030	0.259
	1 to 60 d	1.68	1.66	1.71	1.70	0.049	0.757
F:G ^5^	1 to 15 d	6.72	6.42	7.30	7.73	1.377	0.785
	16 to 30 d	11.02	14.36	16.27	15.89	2.548	0.232
	31 to 45 d	16.14	14.79	9.35	9.56	2.568	0.060
	46 to 60 d	9.79	8.05	8.14	7.82	2.443	0.842
	1 to 60 d	10.87	10.97	10.80	10.88	1.100	0.999

^1^ SEM = standard error of the mean; ^2^ BW = body weight; ^3^ ADG = average daily gain; ^4^ ADFI = average daily feed intake; ^5^ F:G = feed-to-gain ratio. XOS0% = basal diet; XOS1.5% group = basal diet + 1.5 g/kg XOS; XOS3% group = basal diet + 3 g/kg XOS; XOS4.5% group = basal diet + 4.5 g/kg XOS. In the same row, values with no letters or the same letter superscripts indicate no significant difference (*p* > 0.05), while different small letter superscripts indicate significant difference (*p* < 0.05).

**Table 3:** Effects of XOS on body measurement index of Hu lambs.

Item	Group				SEM ^1^	*p*-Value
	XOS0%	XOS1.5%	XOS3%	XOS4.5%		
1 d						
Body Length Index (%)	101.52	95.08	95.15	102.39	3.140	0.084
Body Trunk Index (%)	112.60	112.68	113.18	108.57	3.021	0.440
Chest Circumference Index (%)	114.21 ^a^	107.00 ^b^	107.66 ^b^	111.16 ^ab^	2.328	0.048
Metacarpal Circumference Index (%)	12.67	12.77	12.26	13.19	0.418	0.252
15 d						
Body Length Index (%)	94.79	98.85	92.17	94.90	4.186	0.497
Body Trunk Index (%)	108.67	102.38	104.00	105.66	3.432	0.362
Chest Circumference Index (%)	102.75	101.15	95.81	100.24	3.380	0.276
Metacarpal Circumference Index (%)	12.85	12.37	12.76	12.67	0.914	0.956
30 d						
Body Length Index (%)	96.14	96.24	100.03	101.67	3.904	0.437
Body Trunk Index (%)	104.98	100.11	100.86	105.05	2.935	0.262
Chest Circumference Index (%)	100.90	96.26	100.86	106.72	3.626	0.109
Metacarpal Circumference Index (%)	12.56	12.35	11.39	12.07	1.027	0.694
45 d						
Body Length Index (%)	99.01	103.47	101.74	104.49	3.852	0.540
Body Trunk Index (%)	98.75	96.88	98.48	102.64	4.549	0.646
Chest Circumference Index (%)	97.64	100.12	99.99	107.32	4.250	0.201
Metacarpal Circumference Index (%)	11.86	12.15	12.75	13.09	0.630	0.273
60 d						
Body Length Index (%)	98.95 ^b^	98.45 ^b^	103.1 ^ab^	105.59 ^a^	2.213	0.034
Body Trunk Index (%)	106.06	104.34	98.51	105.26	2.561	0.066
Chest Circumference Index (%)	104.97	102.76	101.54	111.10	3.368	0.085
Metacarpal Circumference Index (%)	12.37	13.06	12.18	12.76	0.370	0.157

^1^ SEM = standard error of the mean. XOS0% = basal diet; XOS1.5% group = basal diet + 1.5 g/kg XOS; XOS3% group = basal diet + 3 g/kg XOS; XOS4.5% group = basal diet + 4.5 g/kg XOS; a–b, in the same row, mean values with different superscripts were significantly different (*p* < 0.05).

**Table 4:** Effects of XOS on carcass traits of Hu lambs.

Item	Group				SEM ^1^	*p*-Value
	XOS0%	XOS1.5%	XOS3%	XOS4.5%		
pre-slaughter weight/kg	27.20	27.30	28.23	27.72	0.907	0.669
net meat weight/kg	9.40	9.40	9.37	9.23	0.267	0.909
bone weight/kg	2.53	2.57	2.80	2.53	0.153	0.307
carcass weight/kg	12.02	12.04	12.25	11.85	0.328	0.702
slaughter rate/%	44.19	44.19	43.40	42.86	2.039	0.890
meat yield/%	34.59	34.49	33.21	33.40	1.753	0.800
meat-to-bone ratio/%	26.93	27.31	29.93	27.48	1.711	0.347
tail fat/g	295.00	231.67	257.00	305.33	27.793	0.094
Longissimus dorsi muscle area/cm^2^	15.67	15.49	14.57	13.74	1.385	0.515
GR value/mm	5.86 ^C^	6.84 ^A^	6.60 ^B^	6.84 ^A^	0.077	<0.01
rumen pH	5.48 ^B^	5.65 ^B^	5.98 ^A^	6.01 ^A^	0.084	<0.01

^1^ SEM = standard error of the mean. XOS0% = basal diet; XOS1.5% group = basal diet + 1.5 g/kg XOS; XOS3% group = basal diet + 3 g/kg XOS; XOS4.5% group = basal diet + 4.5 g/kg XOS; A–C, In the same row, mean values with different superscripts were highly significantly different (*p* < 0.01).

**Table 5:** Effects of XOS on mutton quality characteristics.

Item	Group				SEM ^1^	*p*-Value
	XOS0%	XOS1.5%	XOS3%	XOS4.5%		
Moisture content (%)	77.77	76.87	77.47	78.40	1.363	0.732
Fat content (%)	1.30 ^a^	1.07 ^b^	1.17 ^a^	0.80 ^b^	0.145	0.045
Muscle pH	5.79	5.59	5.89	5.57	0.125	0.093
Cooking loss (%)	36.41	37.91	37.08	34.84	1.806	0.429
Shear force (N)	43.12	40.97	41.96	44.56	1.684	0.247
L*	15.73	13.58	15.71	14.66	1.168	0.278
a*	4.80	5.51	4.01	4.78	0.726	0.304
b*	6.13	6.24	6.81	6.56	0.415	0.398
Crude protein (%)	20.62	19.50	19.35	19.53	0.838	0.447

^1^ SEM = standard error of the mean. XOS0% = basal diet; XOS1.5% group = basal diet + 1.5 g/kg XOS; XOS3% group = basal diet + 3 g/kg XOS; XOS4.5% group = basal diet + 4.5 g/kg XOS; a–b, In the same row, mean values with different superscripts were significantly different (*p* < 0.05).

**Table 6:** Effects of XOS on mutton textural properties.

Item	Group				SEM ^1^	*p*-Value
	XOS0%	XOS1.5%	XOS3%	XOS4.5%		
Hardness (g)	8876.55	11,757.42	11,812.01	12,507.73	2791.023	0.596
Adhesiveness (g*s)	−6.99	−2.37	−4.57	−0.61	4.532	0.557
Springiness	0.46	0.56	0.47	0.54	0.046	0.141
Cohesiveness	0.58	0.55	0.54	0.55	0.040	0.850
Gumminess (g)	5178.17	6609.15	6424.72	6999.35	1878.370	0.790
Chewiness (g)	2430.51	3730.20	3060.65	3692.11	1086.358	0.611
Resilience	0.28	0.26	0.26	0.27	0.031	0.934

^1^ SEM = standard error of the mean. XOS0% = basal diet; XOS1.5% group = basal diet + 1.5 g/kg XOS; XOS3% group = basal diet + 3 g/kg XOS; XOS4.5% group = basal diet + 4.5 g/kg XOS; In the same row, values with no letter or the same letter superscripts indicate no significant difference (*p* > 0.05), while different letter superscripts indicate significant difference (*p* < 0.05).

## Data Availability

The original contributions presented in this study are included in the article. Further inquiries can be directed to the corresponding author.
